# Androgenetic alopecia drugs and male infertility: Evidence from pharmacovigilance and testicular transcriptomics

**DOI:** 10.1371/journal.pone.0357154

**Published:** 2026-09-24

**Authors:** Zhuozhi Gong, Jing He, Qiujian Feng, Wenyu Chen, Yonglong Xu, Qingying Wang, Shengjing Liu

**Affiliations:** 1 Department of Andrology, Xiyuan Hospital of China Academy of Chinese Medical Sciences, Beijing, China; 2 School of Traditional Chinese Medicine, Southern Medical University, Guangzhou, China; 3 Beijing University of Chinese Medicine, Beijing, China; 4 Department of Dermatology, Xiyuan Hospital of China Academy of Chinese Medical Sciences, Beijing, China; University of Veterinary and Animal Sciences, PAKISTAN

## Abstract

**Objective:**

To identify disproportionality signals linking androgenetic alopecia (AGA) drugs (finasteride, dutasteride, and minoxidil) with male infertility-related adverse events and to explore infertility-related biological features using testicular transcriptomic datasets.

**Methods:**

A dual-database replication design was employed using FAERS (2004–2025) and EudraVigilance (2002–2025). Disproportionality analysis with multiple signal detection metrics assessed drug–infertility associations. Multi-level bioinformatic analyses—including toxicity prediction, drug-associated target screening, testicular transcriptomic analysis, single-cell RNA sequencing, intercellular communication analysis, gene set enrichment analysis (GSEA), and immune infiltration analysis—were integrated to explore biological features potentially relevant to male infertility.

**Results:**

Disproportionality analyses detected signals for all three drugs, with finasteride demonstrating the most prominent reporting signal, followed by dutasteride and minoxidil. Multi-level bioinformatic analysis identified HIF1A as an overlap-derived candidate under the specified datasets and screening criteria, and HIF1A expression was higher in testicular tissue from patients with male infertility. Single-cell analysis showed higher HIF1A expression in late spermatocytes, Leydig cells, and myoid cells from infertility samples, together with group-dependent inferred communication patterns for selected VEGF-, PDGF-, IGF-, and FGF-related signaling axes. GSEA associated higher HIF1A expression with immune-response-, wound-healing-, and cell-adhesion-related processes, whereas lower HIF1A expression was associated with spermatid-development- and cilium/flagellum-dependent motility-related processes. ssGSEA-based analysis showed positive correlations between HIF1A expression and several immune-cell signature scores.

**Conclusion:**

This study systematically evaluated associations between AGA drugs and male infertility using real-world pharmacovigilance data and integrated bioinformatic analyses. The HIF1A-related transcriptomic findings provide a hypothesis-generating biological context for male infertility but do not establish a shared or drug-specific mechanism linking the three medications to infertility. The pharmacovigilance findings indicate reporting signals rather than incidence or causality.

## 1. Introduction

Androgenetic alopecia (AGA) is the most common type of hair loss. Epidemiological studies indicate that approximately 30%–50% of men develop varying degrees of AGA before age 50, with a lifetime risk reaching up to 80% [1]. Notably, recent large-scale cohort studies suggest a trend toward younger age at treatment-seeking among male AGA patients, predominantly concentrated in the 20–39 age group [2]. Current standard pharmacological treatments for AGA primarily include oral 5α-reductase inhibitors (finasteride and dutasteride) and minoxidil formulations (topical solutions or foams, with low-dose oral regimens also adopted in some countries/regions), which are consistently recognized in multiple national guidelines and expert consensus as the first-line therapies with the most substantial evidence [3]. Among these agents, oral finasteride at 1 mg daily has been approved by the U.S. Food and Drug Administration (FDA) for AGA treatment, while dutasteride, although not yet approved by the U.S. FDA for this indication, has been licensed in countries such as South Korea and Japan [4]. With the rising prevalence of AGA and increasing cosmetic demands related to hair loss, the prescription volume and market size of AGA therapeutic agents, represented by finasteride, dutasteride, and minoxidil, continue to expand globally, with estimated annual market revenues exceeding 4 billion U.S. dollars [5]. Given that AGA predominantly affects males and is highly prevalent among men aged 20–50 years during their reproductive years, a substantial number of men of childbearing age are receiving long-term treatment with these medications, with finasteride and dutasteride being primarily prescribed to reproductive-age males between 20–40 years in clinical practice [6,7]

In stark contrast to the expanding use of AGA therapeutic agents, male fertility worldwide is experiencing a persistent declining trend, which continues to accelerate in the 21st century [8]. Epidemiological data reveal that approximately 15% of couples of reproductive age globally face infertility issues, with nearly half of these cases being attributed to male factors [9]. Data analysis from the Global Burden of Disease Study demonstrates that the number of male infertility patients worldwide exceeded 56.5 million in 2019, representing an approximately 77% increase compared to 1990, with age-standardized prevalence rates showing a continuous upward trend in most regions, particularly concentrated among males aged 20–44 years during their peak reproductive period [10]. The decline in male fertility not only impacts family reproduction but may also exert profound effects on societal demographic structures. The etiology of male infertility is complex and multifaceted, encompassing genetic factors, endocrine abnormalities, reproductive system infections, environmental exposures, and pharmacological factors [11–13].

However, regarding the impact of AGA therapeutic agents on male fertility, existing clinical research conclusions are contradictory, with evidence quality being highly variable. Finasteride and dutasteride, as 5α-reductase inhibitors, exert their therapeutic effects by inhibiting the conversion of testosterone to dihydrotestosterone (DHT), but their systemic actions may affect reproductive axis function. Existing research findings exhibit marked heterogeneity: some prospective clinical trials have reported no clear changes in semen parameters; for example, a 48-week study in healthy men receiving oral finasteride 1 mg daily reported no clear changes in sperm concentration, total count, motility, or morphology [14]. However, other studies have reported opposite results, including decreases in semen volume, sperm count, concentration, and motility [15], with recovery of sperm counts after drug discontinuation [16], suggesting that this agent may exert reversible inhibitory effects on spermatogenesis in some patients. Dutasteride, as a more potent 5α-reductase inhibitor (simultaneously inhibiting both type I and type II enzymes), presents more complex reproductive toxicity evidence: some studies report total sperm count declining to below 10% of baseline during treatment, with partial recovery after discontinuation [15]; however, long-term follow-up studies have also identified persistent semen quality impairment [7]. Compared to 5α-reductase inhibitors, research on minoxidil's effects on the male reproductive system is relatively scarce. Conventional perspectives consider topical minoxidil to have relatively high reproductive safety; however, a systematic review indicates that minoxidil can act as an endocrine disruptor, inducing oxidative stress and morphological damage in the testes [17]. Overall, research reports on the relationship between these three agents and male infertility remain limited, particularly with insufficient data support for the potential reproductive effects of dutasteride and minoxidil, and their specific impacts on clinical fertility remain controversial.

Given the persistently increasing burden of male infertility, the widespread use of AGA therapeutic agents among reproductive-age males, contradictory clinical evidence, and the lack of mechanistic research, systematic evaluation of the association between these medications and male infertility and elucidation of their underlying mechanisms hold clinical and public health value. This study adopts a dual-database cross-validation strategy based on real-world data from the FDA Adverse Event Reporting System (FAERS) and the European Union pharmacovigilance database (EudraVigilance), employing disproportionality analysis to assess the signal strength of reported associations between finasteride, dutasteride, and minoxidil and male infertility-related adverse events, thereby addressing limitations of existing clinical research. Building on the pharmacovigilance analysis, we further integrated toxicity prediction, drug-associated target screening, bulk transcriptomics, single-cell RNA sequencing, and intercellular communication analysis to explore biological features potentially relevant to male infertility. These analyses were intended to generate hypotheses rather than to establish drug-specific mechanisms.

## 2. Methods

### 2.1. Pharmacovigilance data source and processing

Data for this study were obtained from the FDA Adverse Event Reporting System (FAERS; Quarter 1, 2004 to Quarter 2, 2025) and the European Union pharmacovigilance database (EudraVigilance, EV; January 2002 to October 2025). FAERS data were deduplicated in accordance with official recommendations. The PRIMARYID, CASEID, and FDA_DT fields were extracted from the DEMO table, and the most recent report version was retained through three-level sorting. Withdrawn reports were excluded from Quarter 1, 2019 onward. Adverse events were standardized at the Preferred Term (PT) and System Organ Class (SOC) levels using the Medical Dictionary for Regulatory Activities (MedDRA), version 28.0, and drug names were normalized using the March 2025 edition of the World Health Organization Drug Dictionary. To account for potential changes in terminology and hierarchy across MedDRA versions, historical records were remapped using the current dictionary version.

The target drugs were finasteride, dutasteride, and minoxidil, selected because of their established clinical use in AGA treatment. Given the substantial missingness and inconsistent coding of indication information, pharmacovigilance analyses were conducted at the drug level. Available dose and administration-route information was summarized descriptively.

Target adverse events comprised male infertility–related events identified using 13 MedDRA PTs (Table 1). For FAERS, the analysis was restricted to reports in which patient sex was recorded as male (“M” in the DEMO table); reports with missing or unknown sex were excluded. For EudraVigilance, the analysis was similarly restricted to reports recorded as male, and reports with unknown sex were excluded. EudraVigilance data were extracted from the publicly accessible ADR website on 27 October 2025.

**Table 1:** MedDRA preferred terms used to identify male infertility–related adverse events.

Preferred Term Code	Preferred Term
10050208	Teratospermia
10030300	Oligospermia
10067162	Asthenospermia
10041493	Spermatogenesis abnormal
10021929	Infertility male
10079294	Oligoasthenoteratozoospermia
10080320	Oligoasthenozoospermia
10074268	Reproductive toxicity
10074729	Necrospermia
10003495	Aspermia
10003883	Azoospermia
10049572	Testicular necrosis
10043298	Testicular atrophy

To minimize uncertainty in drug attribution, exposure was defined as Primary Suspect (PS) in FAERS and Suspected in EudraVigilance, and only reports meeting these criteria were included. When multiple suspect drugs were listed in the same report, each drug–event pair contributed to the corresponding 2 × 2 contingency table. The primary analysis retained reports involving concomitant medications, whereas the sensitivity analysis was restricted to reports without concomitant medications.

Indication- and administration-route-stratified analyses were not performed because the completeness of these fields was insufficient to support reliable subgroup analyses.

As these datasets (FAERS and EudraVigilance) are fully de-identified and publicly available, no ethics approval or informed consent was required for this study.

### 2.2. Pharmacovigilance signal detection and sensitivity analysis

A 2 × 2 contingency table was constructed to calculate drug-adverse event association metrics (S1 Table). Signal detection was performed using four combined methods: Reporting Odds Ratio (ROR), Proportional Reporting Ratio (PRR), Bayesian Confidence Propagation Neural Network (BCPNN), and Multi-item Gamma Poisson Shrinker (MGPS), with calculation formulas and thresholds detailed in S2 Table. A positive signal was defined as simultaneously meeting the following criteria: adverse event report count ≥3, lower limit of 95% confidence interval (CI) of ROR > 1, PRR ≥ 2 and χ² ≥ 4, information component (IC) minus 2 standard deviations (SD) >0, and empirical Bayes geometric mean 5th percentile (EB05) >2 [18]. ROR values were used to quantify reporting disproportionality, with larger values indicating greater disproportionality within the database; they were not interpreted as estimates of incidence or comparative risk across drugs. A sensitivity analysis restricted to reports without concomitant medications was performed. Disproportionality metrics were recalculated to assess the stability of the primary findings. Disproportionality metrics quantify disproportionate reporting in spontaneous reporting systems and should be interpreted as hypothesis-generating safety signals rather than estimates of incidence or causal effects.

### 2.3. Drug toxicity prediction and common target screening

To generate biological hypotheses relevant to the pharmacovigilance signals, this study integrated multi-level bioinformatic approaches to explore biological features associated with male infertility. These analyses were not designed to establish drug-specific mechanisms. Chemical structures and Simplified Molecular Input Line Entry System (SMILES) strings of the three drugs were obtained from PubChem (https://pubchem.ncbi.nlm.nih.gov/). ProTox3.0 (https://tox.charite.de/protox3/) was employed to predict toxicity risks to different tissues and organs based on chemical structures, utilizing a consensus method integrating 2D similarity, fragment propensity, and target binding probability. Admetlab3.0 (https://admetlab3.scbdd.com/) was used for TOX21 PATHWAY analysis to predict compound effects on 12 nuclear receptor signaling pathways and stress response pathways. Drug-associated target lists were compiled from CTD, GeneCards, and DrugBank (retrieved on 2025-12-02). These resources contain curated targets and/or evidence-based gene–chemical associations and may include predicted or indirect relationships; therefore, the compiled lists were treated as “drug-associated targets” for hypothesis generation rather than definitive direct binding targets. All retrieved entries were merged and deduplicated to generate a target set for each drug, and the intersection across the three drugs was used for downstream overlap analyses.

### 2.4. Testicular transcriptome differential analysis and overlap-derived candidate identification

The GSE45885 and GSE45887 datasets (GPL6244 platform) were downloaded from the Gene Expression Omnibus (GEO, https://www.ncbi.nlm.nih.gov/gds/) database, including testicular biopsy specimens from 43 male infertility patients and 8 normal controls. Multiple probes for the same gene were averaged using the avereps function in the limma package, retaining single gene expression values. Following log2 transformation and quantile normalization, batch effects were corrected using the ComBat function in the sva package. Linear models were constructed using the limma package to compare inter-group differences, with variance adjusted through eBayes and false discovery rate (FDR) controlled by the Benjamini-Hochberg method. Multiple testing was addressed using the Benjamini–Hochberg procedure, and genes showing statistically supported expression differences were retained for downstream analysis. Intersection analysis between infertility-associated DEGs and the aggregated drug-associated target lists identified overlap-derived candidate genes. Candidate-gene expression patterns were further examined in the normalized dataset using two-sided statistical comparisons, and receiver operating characteristic (ROC) curves were generated to describe dataset-level discriminatory performance.

All bulk transcriptomic analyses were performed in R 4.4.3 using limma v3.62.2 for differential expression modeling, sva v3.54.0 (ComBat) for batch-effect correction, and pROC v1.18.5 for ROC/AUC estimation.

### 2.5. Single-cell transcriptomic analysis

Single-cell transcriptomic analysis was performed using the Seurat package (v4.0) based on the GSE149512 dataset. Cells with 200–6,000 detected genes and 500–30,000 unique molecular identifier (UMI) counts were retained for downstream analysis. Quality-controlled data were normalized using the LogNormalize method (normalization factor = 10,000), and 3000 highly variable genes were selected for principal component analysis (PCA) dimensionality reduction. After data integration and reduction of technical variation using the Harmony algorithm, Uniform Manifold Approximation and Projection (UMAP) was performed based on the first 30 principal components, followed by Louvain clustering at a resolution of 0.6. Cell type annotation referenced previous human testicular single-cell transcriptome atlases (S3 Table) [19–21]. Cells were classified into control and disease groups according to sample grouping information.

Candidate-gene expression values were extracted from normalized data, and their expression distributions were visualized in UMAP space. The Wilcoxon rank-sum test was employed to compare candidate-gene expression differences between the male infertility group and control group, with analyses conducted at both the whole-sample level and major cell type levels. Only cell types with cell counts ≥3 in both groups were included, with adjusted *P* < 0.05 used as the decision threshold. Single-cell preprocessing, clustering, and visualization were conducted using Seurat v4.0 with Harmony-based batch correction implemented via harmony v1.2.3.

### 2.6. Cell communication analysis

Intercellular communication patterns were analyzed using CellChat (v1.6.0) and the CellChatDB.human database. Seurat objects were stratified by group and converted to CellChat objects with cell type annotations. Overexpressed ligands and receptors were identified, communication probabilities were calculated using the triMean method, and communication events involving at least 10 cells were filtered. Communication probabilities were aggregated at the pathway level to obtain overall communication strength for each signaling pathway. Based on research evidence regarding hypoxia response and angiogenesis-related pathways in testicular microenvironment remodeling, particular attention was paid to classical signaling pathways including vascular endothelial growth factor (VEGF), fibroblast growth factor (FGF), platelet-derived growth factor (PDGF), insulin-like growth factor (IGF), erythropoietin (EPO), angiopoietin (ANGPT), and hepatocyte growth factor (HGF). Group-specific communication networks were inferred using consistent analytical settings and were descriptively compared between the control and infertility groups.

### 2.7. Functional enrichment and immune infiltration analysis

For all samples in the merged GSE45885 and GSE45887 dataset, Pearson correlation analysis was performed between candidate-gene expression levels and all genes in the expression matrix. Genes with |r| > 0.6 and FDR < 0.01 were considered correlated with the candidate genes. Gene Ontology (GO) and Kyoto Encyclopedia of Genes and Genomes (KEGG) enrichment analyses were performed on genes correlated with the candidate genes, with *P* < 0.05 and adjusted *P* < 0.05 used as thresholds for enrichment results.

Samples were divided into high- and low-expression groups based on the median candidate-gene expression level. Differential expression analysis was performed to obtain log2 fold change (logFC) values for all genes. After converting gene symbols to ENTREZ IDs, a ranked gene list was constructed in descending order of logFC values. Gene Set Enrichment Analysis (GSEA) for GO biological processes and KEGG pathways was performed using the gseGO and gseKEGG functions in the clusterProfiler package, with analysis parameters set as: minGSSize = 10, maxGSSize = 500, *P* = 0.05. Multiple testing correction was performed using the Benjamini-Hochberg method. Enrichment results were ranked by normalized enrichment score (NES), and the top 5 pathways with the highest absolute NES values in both high-expression and low-expression groups were selected for visualization.

The single-sample Gene Set Enrichment Analysis (ssGSEA) method in the Gene Set Variation Analysis (GSVA) package was used to estimate immune cell infiltration levels for each sample in the merged GSE45885 and GSE45887 dataset. Based on literature-reported immune cell-specific marker gene sets (S4 Table), ssGSEA scores were calculated for each immune cell type, with higher scores indicating greater relative enrichment of the corresponding immune-cell gene signature in the sample [22–24]. Spearman rank correlation analysis was used to assess associations between candidate-gene expression and immune cell infiltration levels, with Benjamini–Hochberg correction applied and FDR < 0.01 used as the decision threshold. Enrichment analyses were performed using clusterProfiler v4.14.6, and ssGSEA-based immune infiltration estimation was conducted using GSVA v2.0.7.

## 3. Results

### 3.1. Baseline characteristics of real-world reporting population

The initial FAERS extraction contained 23,168,942 case-report records. Following the FDA-recommended deduplication procedure, 3,916,613 duplicate or earlier report versions were removed, leaving 19,252,329 unique case reports. These case reports contained 57,212,790 adverse-event records, as a single case report could include multiple adverse events. After application of the prespecified eligibility criteria, 6,701,047 unique case reports, comprising 19,344,257 adverse-event records, were included in the analytic dataset. The EudraVigilance database included a total of 11,092,268 patient reports.

**Fig 1:**
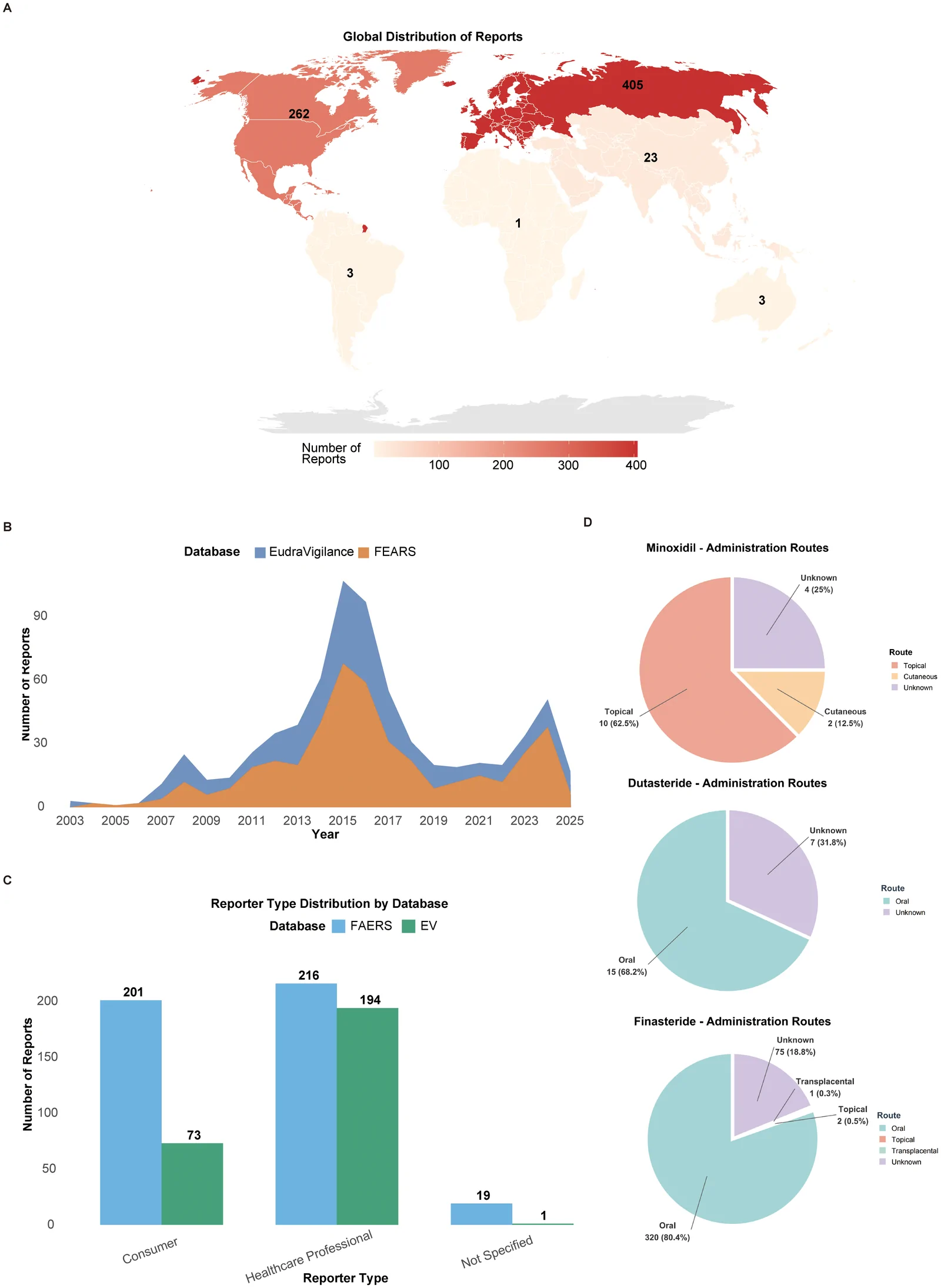
Descriptive characteristics of male infertility–related reports in FAERS and EudraVigilance. A. Global geographical distribution of reports with available location information. The base map in panel A was generated using public-domain Natural Earth vector data accessed through the rnaturalearth R package. B. Annual numbers of male infertility–related reports in FAERS and EudraVigilance. Data for 2025 represent incomplete reporting periods. C. Distribution of reporter types in FAERS and EudraVigilance. D. Distribution of reported administration routes for finasteride, dutasteride, and minoxidil in FAERS.

Following secondary screening, eligible male infertility-related reports were identified in both databases. Geographically, reports were concentrated mainly in Europe and North America (Fig 1A). The annual number of reports varied over the study period, with noticeable fluctuations in both databases (Fig 1B). Consumers and healthcare professionals constituted the main reporter groups in FAERS, whereas healthcare professionals were more prominent in EudraVigilance (Fig 1C). In FAERS, administration-route patterns differed among the three target drugs. Finasteride and dutasteride were predominantly reported with oral administration, whereas minoxidil was mainly reported with topical or cutaneous administration; route information was unavailable for a proportion of reports (Fig 1D).

### 3.2. Dual-database replication of disproportionality signals between three aga therapies and male infertility–related adverse event reports

Multiple disproportionality methods were applied to evaluate disproportionate reporting signals between the three AGA therapies and male infertility–related adverse event reports (Table 2). In the FAERS database, finasteride exhibited the strongest signal intensity, with 469 reports (ROR 73.90, 95% CI 66.70–81.87). Dutasteride had 22 reports (ROR 12.74, 95% CI 8.37–19.39), and minoxidil had 17 reports (ROR 3.30, 95% CI 2.04–5.31). Validation analysis in the EudraVigilance database revealed trends similar to those in FAERS. Finasteride had 288 reports (ROR 60.07, 95% CI 53.03–68.04), dutasteride had 16 reports (ROR 14.64, 95% CI 8.95–23.96), and minoxidil had 11 reports (ROR 9.50, 95% CI 5.25–17.20). The two independent databases showed consistent disproportionality signals for the three drugs with male infertility–related adverse event reports, supporting the robustness of these reporting signals.

**Table 2:** Disproportionality analysis of male infertility–related adverse-event reports for the three target drugs.

	FAERS						EV					
Drug name	n	ROR(95% CI)	PRR(95% CI)	χ²	IC(IC025)	EB(EB05)	n	ROR(95% CI)	PRR(95% CI)	χ²	IC(IC025)	EB(EB05)
Dutasteride	22	12.74(8.37, 19.39)	12.72(8.36, 19.36)	235.22	3.66(2.46)	12.60(8.28)	16	14.64(8.95, 23.96)	14.62(8.94, 23.90)	201.51	3.86(2.31)	14.52(8.87)
Finasteride	469	73.90(66.70, 81.87)	73.43(66.31, 81.31)	26276.9	5.85(5.54)	57.79(52.17)	288	60.07(53.03, 68.04)	59.68(52.73, 67.55)	14365.3	5.69(5.28)	51.72(45.66)
Minoxidil	17	3.30(2.04, 5.31)	3.29(2.04, 5.31)	26.95	1.71(0.86)	3.28(2.03)	11	9.50(5.25, 17.20)	9.49(5.25, 17.16)	83.18	3.24(1.64)	9.45(5.22)

### 3.3. Testicular atrophy and sperm abnormalities constitute the predominant reported event spectrum in male infertility–related reports involving AGA therapies

To further characterize the male infertility–related adverse-event types reported for the three target drugs, this study conducted subgroup disproportionality analysis stratified by adverse reaction type based on 13 predefined MedDRA PTs (Table 3). Testicular atrophy was a commonly reported adverse reaction across all three drugs. Finasteride-associated testicular atrophy had the highest number of reports, totaling 224 reports (ROR 112.04, 95% CI 95.84–130.98). Dutasteride had 11 reports (ROR 18.30, 95% CI 10.09–33.20). Minoxidil had 11 reports (ROR 6.15, 95% CI 3.39–11.15). Regarding impaired fertility, finasteride-associated male infertility comprised 68 reports (ROR 51.15, 95% CI 39.46–66.31). Dutasteride had 4 reports (ROR 11.88, 95% CI 4.44–31.80). No positive disproportionality signal for the PT “Infertility male” was identified for minoxidil. Sperm abnormalities were primarily observed with finasteride and dutasteride. Concerning sperm quantity, finasteride-associated azoospermia comprised 32 reports (ROR 26.20, 95% CI 18.22–37.67), oligospermia comprised 23 reports (ROR 97.43, 95% CI 60.43–157.06), and oligoasthenozoospermia comprised 4 reports (ROR 71.14, 95% CI 23.61–214.37). Dutasteride only showed azoospermia with 4 reports (ROR 14.06, 95% CI 5.25–37.67). Regarding sperm quality, finasteride-associated teratospermia comprised 9 reports (ROR 64.90, 95% CI 31.32–134.47), and asthenospermia comprised 6 reports (ROR 30.78, 95% CI 13.22–71.67). Additionally, finasteride had 6 reports of abnormal spermatogenesis (ROR 21.34, 95% CI 9.29–49.03).

**Table 3:** Preferred-term-level disproportionality signals for male infertility–related adverse events reported with the three target drugs.

Drug name	adverse reaction	n	ROR(95% CI)	PRR(95% CI)	χ²	IC(IC025)	EB(EB05)
Dutasteride	Testicular atrophy	11	18.30(10.09,33.20)	18.29(10.09,33.17)	177.21	4.17(2.06)	18.04(9.95)
Dutasteride	Infertility male	4	11.88(4.44,31.80)	11.87(4.44,31.78)	39.46	3.56(0.60)	11.77(4.40)
Dutasteride	Azoospermia	4	14.06(5.25,37.67)	14.05(5.25,37.65)	47.96	3.80(0.66)	13.91(5.19)
Finasteride	Testicular atrophy	224	112.04(95.84,130.98)	111.70(95.58,130.53)	17322.5	6.30(5.65)	79.03(67.60)
Finasteride	Infertility male	68	51.15(39.46,66.31)	51.10(39.43,66.23)	2803.31	5.43(4.36)	43.05(33.21)
Finasteride	Azoospermia	32	26.20(18.22,37.67)	26.19(18.21,37.65)	705.92	4.58(3.29)	23.94(16.65)
Finasteride	Oligospermia	23	97.43(60.43,157.06)	97.40(60.42,157.00)	1607.48	6.16(3.51)	71.61(44.42)
Finasteride	Teratospermia	9	64.90(31.32,134.47)	64.89(31.32,134.45)	455.40	5.71(2.08)	52.39(25.29)
Finasteride	Asthenospermia	6	30.78(13.22,71.67)	30.78(13.22,71.66)	155.00	4.79(1.37)	27.70(11.90)
Finasteride	Spermatogenesis abnormal	6	21.34(9.29,49.03)	21.34(9.29,49.02)	107.72	4.31(1.29)	19.84(8.64)
Finasteride	Oligoasthenozoospermia	4	71.14(23.61,214.37)	71.14(23.61,214.35)	218.38	5.82(0.78)	56.37(18.71)
Minoxidil	Testicular atrophy	11	6.15(3.39,11.15)	6.14(3.39,11.14)	46.69	2.60(1.25)	6.07(3.35)

### 3.4. Sensitivity analysis in reports without concomitant medications supports the robustness of disproportionality signals

To assess the robustness of the primary findings, sensitivity disproportionality analyses were performed after restricting the dataset to reports without medications coded as concomitant (Table 4). After excluding reports with concomitant medications, dutasteride-associated testicular atrophy was reduced to 7 reports (ROR 29.41, 95% CI 13.93–62.12), with a numerically higher ROR. Male infertility was reduced to 3 reports (ROR 19.15, 95% CI 6.14–59.78), still maintaining strong signal intensity. Sensitivity analysis results for finasteride showed 163 reports of testicular atrophy (ROR 105.01, 95% CI 86.23–127.89) and 57 reports of male infertility (ROR 43.12, 95% CI 32.20–57.76), highly consistent with primary analysis results. Regarding sperm abnormalities, azoospermia comprised 23 reports (ROR 28.11, 95% CI 18.06–43.77), oligospermia comprised 17 reports (ROR 110.53, 95% CI 59.68–204.69), teratospermia comprised 6 reports (ROR 51.32, 95% CI 20.49–128.50), asthenospermia comprised 5 reports (ROR 38.69, 95% CI 14.59–102.61), and abnormal spermatogenesis comprised 5 reports (ROR 19.35, 95% CI 7.65–48.90). In this restricted dataset, the disproportionality estimates for several adverse events were numerically higher than those in the primary analysis. Minoxidil-associated testicular atrophy comprised 10 reports (ROR 5.39, 95% CI 2.88–10.09), highly consistent with primary analysis results.

**Table 4:** Sensitivity disproportionality analysis of male infertility–related adverse events in reports without concomitant medications.

Drug name	adverse reaction	n	ROR(95% CI)	PRR(95% CI)	χ²	IC(IC025)	EB(EB05)
Dutasteride	Testicular atrophy	7	29.41(13.93,62.12)	29.37(13.92,61.97)	188.64	4.85(1.66)	28.90(13.68)
Dutasteride	Infertility male	3	19.15(6.14,59.78)	19.14(6.14,59.70)	51.01	4.24(0.33)	18.94(6.07)
Finasteride	Testicular atrophy	163	105.01(86.23,127.89)	104.69(86.00,127.44)	10181.0	6.00(5.26)	64.06(52.60)
Finasteride	Infertility male	57	43.12(32.20,57.76)	43.08(32.17,57.68)	1851.90	5.10(4.02)	34.26(25.58)
Finasteride	Azoospermia	23	28.11(18.06,43.77)	28.10(18.05,43.74)	512.49	4.59(2.98)	24.10(15.48)
Finasteride	Oligospermia	17	110.53(59.68,204.69)	110.49(59.67,204.60)	1097.94	6.05(3.02)	66.17(35.73)
Finasteride	Teratospermia	6	51.32(20.49,128.50)	51.31(20.49,128.48)	224.95	5.29(1.37)	39.24(15.67)
Finasteride	Asthenospermia	5	38.69(14.59,102.61)	38.69(14.59,102.60)	148.27	4.97(1.06)	31.44(11.85)
Finasteride	Spermatogenesis abnormal	5	19.35(7.65,48.90)	19.34(7.65,48.89)	77.73	4.12(0.96)	17.39(6.88)
Minoxidil	Testicular atrophy	10	5.39(2.88,10.09)	5.39(2.88,10.09)	34.87	2.40(1.04)	5.28(2.82)

### 3.5. HIF1A is identified as an overlap-derived candidate from drug-associated target lists and infertility-related testicular transcriptomics

**Fig 2:**
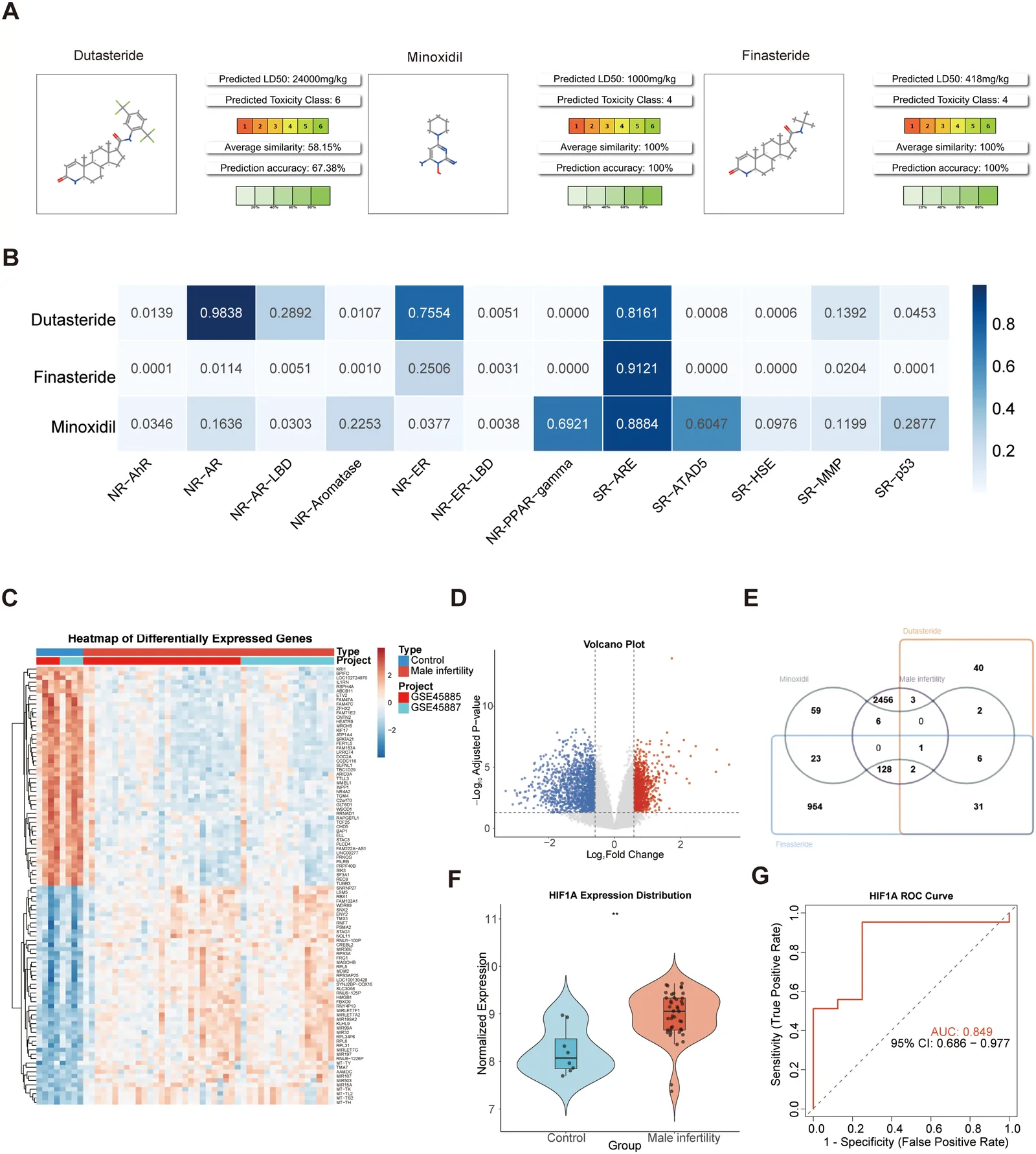
HIF1A is an overlap-derived candidate and shows higher expression in male infertility testicular samples. A. ProTox3.0 prediction of acute toxicity characteristics of three AGA therapeutic agents. B. Heatmap of activation probabilities for three AGA therapeutic agents in TOX21 pathways. C. Heatmap of differentially expressed genes in testicular tissue of male infertility. D. Volcano plot of differentially expressed genes in male infertility. E. Venn diagram of targets of three AGA therapeutic agents and differentially expressed genes in male infertility. F. Violin plot of HIF1A expression levels in control group and male infertility patient group. G. ROC curve showing the exploratory dataset-level discriminatory performance of HIF1A in the analyzed transcriptomic datasets.

Following the identification of consistent disproportionality signals in FAERS and EudraVigilance, we performed in silico toxicity prediction and integrated bioinformatic analyses to explore candidate biological features. ProTox3.0 predicted different acute toxicity profiles for the three drugs, with dutasteride showing lower predicted acute toxicity than finasteride and minoxidil (Fig 2A). These computational predictions reflect general acute toxicity characteristics and should not be interpreted as evidence of reproductive toxicity.

The predicted activity profiles of the three drugs varied across the examined Tox21 nuclear receptor and stress-response endpoints (Fig 2B). Dutasteride showed relatively prominent predicted activity across selected nuclear receptor and stress-response endpoints, whereas finasteride showed a prominent predicted response involving the antioxidant response element. Minoxidil showed relatively high predicted activity across several oxidative-stress- and nuclear-receptor-related endpoints. These computational results describe predicted toxicity-related profiles but do not establish pathway activation or biological effects in testicular tissue.

Integration of CTD, GeneCards, and DrugBank generated drug-associated target lists for the three medications, while differential expression analysis of the GSE45885 and GSE45887 datasets identified a broad set of infertility-associated DEGs (Figs 2C–D). Under the specified databases and screening criteria, intersection analysis prioritized HIF1A as an overlap-derived candidate (Fig 2E). This database-level overlap does not indicate that the investigated drugs directly regulate HIF1A in testicular tissue. HIF1A expression was higher in testicular samples from patients with male infertility than in normal controls (Fig 2F).

ROC analysis suggested that HIF1A had exploratory dataset-level discriminatory performance in the analyzed transcriptomic datasets (Fig 2G). However, this result was derived from the discovery datasets and requires validation in independent cohorts. It should not be interpreted as establishing HIF1A as a diagnostic biomarker or as evidence of its involvement in drug-related reproductive toxicity.

### 3.6. Single-cell transcriptomic analysis of HIF1A expression and intercellular communication in male infertility testicular samples

**Fig 3:**
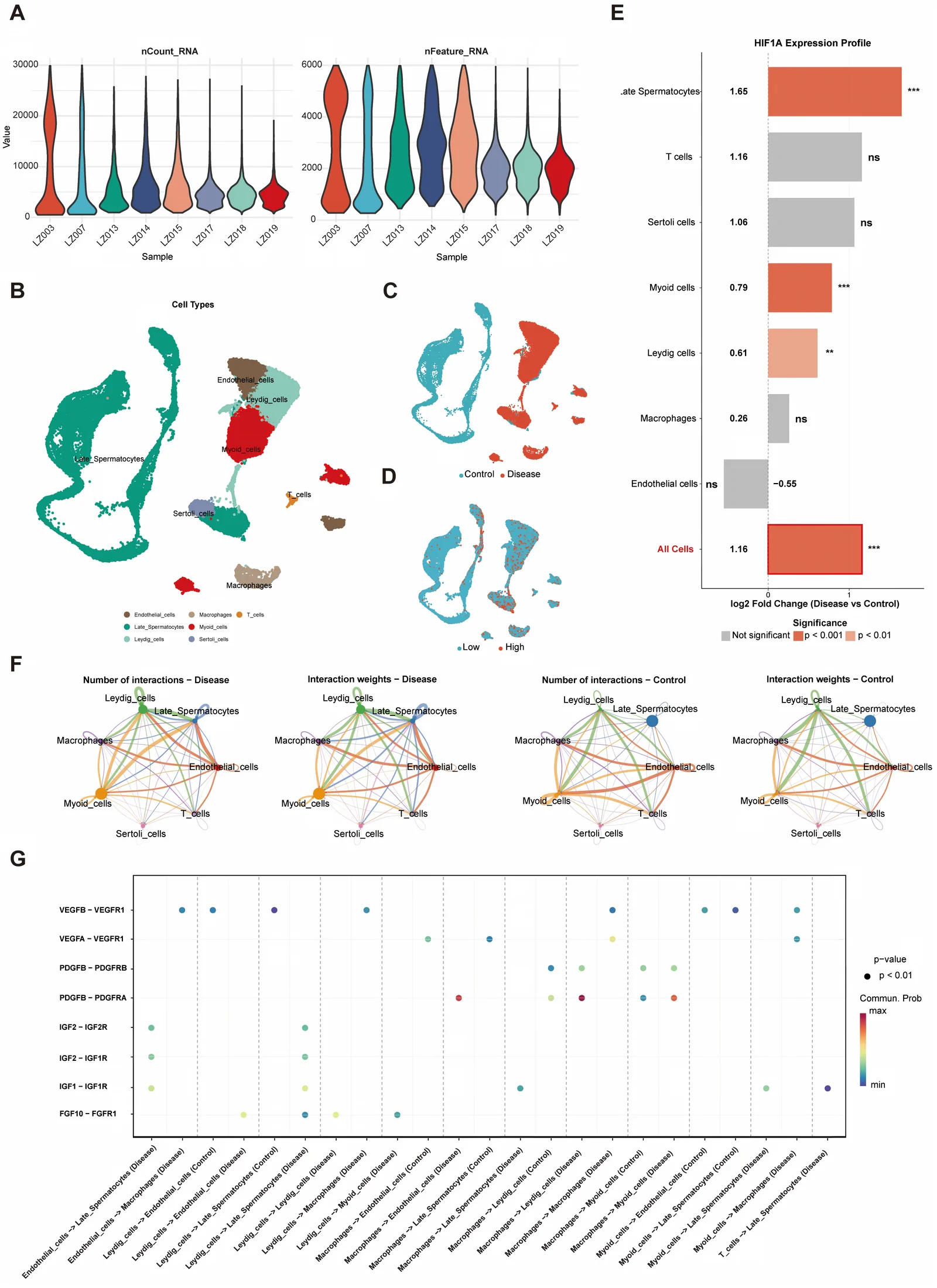
Cell-type-resolved HIF1A expression and growth-factor-related intercellular communication in male infertility testicular samples. A. Violin plot of single-cell RNA sequencing quality control metrics. B. UMAP dimensionality reduction plot of 7 major cell types in testicular tissue based on marker gene annotation. C. UMAP dimensionality reduction visualization of sample grouping. D. UMAP visualization of HIF1A gene expression. E. Bar chart of differential expression analysis of HIF1A in different cell types. F. Comparative analysis of intercellular communication networks between control and disease groups. G. Bubble plot of inferred ligand–receptor communication probabilities across selected angiogenesis- and growth-factor-related signaling axes.

Single-cell transcriptomic analysis was performed using the GSE149512 dataset. Cells retained after filtering based on detected gene numbers and UMI counts were included in the downstream analysis (Fig 3A). Seven major cell types were identified: late spermatocytes, Leydig cells, macrophages, myoid cells, Sertoli cells, T cells, and endothelial cells (Fig 3B). UMAP visualization showed the distribution of cells from the control and infertility groups across the major cell populations (Fig 3C).

HIF1A showed heterogeneous expression across the identified testicular cell populations (Fig 3D). Cell type-specific analysis indicated higher HIF1A expression in late spermatocytes, myoid cells, and Leydig cells in the infertility group, with the most pronounced difference observed in late spermatocytes. No clear between-group differences were observed in T cells, Sertoli cells, macrophages, or endothelial cells (Fig 3E).

CellChat analysis visualized the inferred numbers and weights of intercellular interactions in the control and infertility groups, showing group-dependent differences in the overall communication patterns (Fig 3F). Ligand–receptor analysis further showed group-dependent patterns of inferred communication probability across selected angiogenesis- and growth-factor-related signaling axes. These patterns involved VEGF-, PDGF-, IGF-, and FGF-related ligand–receptor pairs across several cell–cell directions, particularly those involving late spermatocytes (Fig 3G). These CellChat-derived results are inferential and do not establish direct biological communication or HIF1A-mediated regulation.

### 3.7. HIF1A-correlated transcriptomic features and immune-cell signatures in male infertility

**Fig 4:**
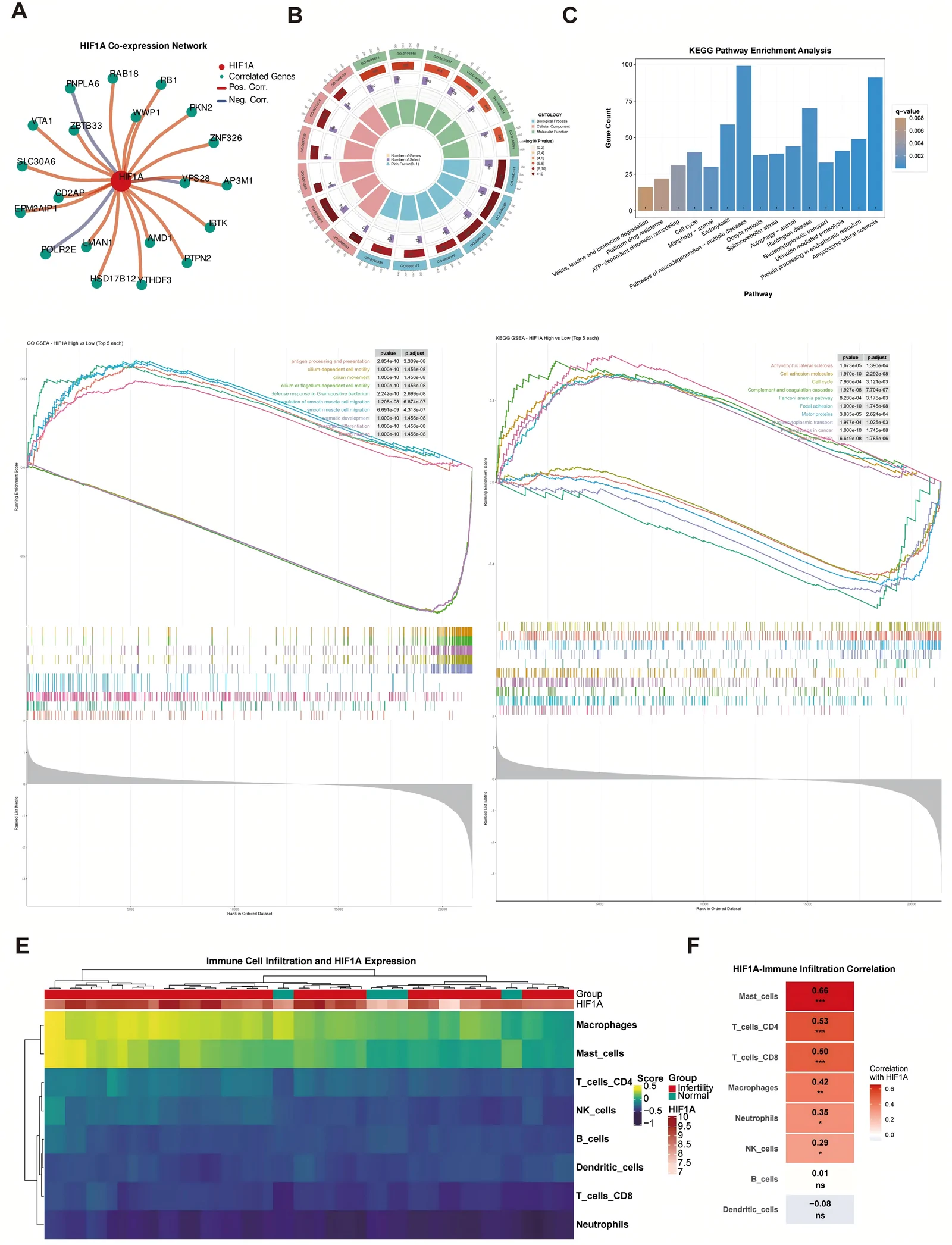
HIF1A-correlated transcriptomic and immune-cell signature features in male infertility testicular transcriptomes. A. Network of genes positively and negatively correlated with HIF1A expression. B. Circular visualization of Gene Ontology enrichment results for HIF1A-correlated genes. C. KEGG pathway enrichment analysis of HIF1A-correlated genes. D. Gene set enrichment analysis comparing the HIF1A-high and HIF1A-low groups. E. Heatmap of ssGSEA-derived immune-cell signature scores across the analyzed samples. F. Spearman correlations between HIF1A expression and ssGSEA-derived immune-cell signature scores.

Correlation analysis of the merged GSE45885 and GSE45887 datasets identified a broad set of genes associated with HIF1A expression (S5 Table). The co-expression network visualized positive and negative correlations between HIF1A and its most strongly correlated genes (Fig 4A). GO and KEGG enrichment analyses indicated that HIF1A-correlated genes were associated with diverse cellular, metabolic, mitochondrial, and protein-homeostasis-related processes (Figs 4B–C). These enrichment results describe transcriptomic associations and do not establish direct biological functions of HIF1A.

To further characterize transcriptomic differences associated with HIF1A expression, GSEA was performed after stratifying the samples according to HIF1A expression. The HIF1A-high group showed enrichment of immune-response-, wound-healing-, and cell-adhesion-related processes, whereas the HIF1A-low group showed enrichment of processes related to spermatid development, spermatid differentiation, and cilium- or flagellum-dependent motility (Fig 4D).

ssGSEA showed heterogeneous immune-cell signature patterns across the analyzed testicular samples (Fig 4E). After multiple-testing correction, positive correlations meeting the prespecified threshold were observed between HIF1A expression and the signature scores of mast cells, CD4 + T cells, CD8 + T cells, and macrophages. No clear correlations with B-cell or dendritic-cell signatures were observed after correction (Fig 4F).

## 4. Discussion

Using FAERS and EudraVigilance data, we detected fertility-related reporting signals for finasteride, dutasteride, and minoxidil. Finasteride showed the most prominent signal, followed by dutasteride, whereas minoxidil generated fewer reports. These patterns warrant further evaluation; however, spontaneous-reporting data cannot determine incidence, comparative risk, or causality.

Previous studies provide clinical context for the finasteride signal. Reproductive and sexual adverse events have been reported more frequently with finasteride than with minoxidil among men treated for androgenetic alopecia [25]. In men with fertility difficulties, semen volume and sperm concentration improved after finasteride discontinuation [16], suggesting that semen-parameter changes may be reversible in some patients. However, these clinical observations do not validate the pharmacovigilance signal or establish causality.

Clinical studies have also raised concerns about dutasteride, reporting reductions in sperm count, semen volume, and motility during treatment, with incomplete recovery in some participants after discontinuation [15,26]. These findings are biologically compatible with inhibition of testosterone-to-DHT conversion and potential disruption of androgen-dependent spermatogenic processes [26]. Nevertheless, they provide clinical and mechanistic context only and do not explain or confirm the reporting signal observed in this study.

Minoxidil primarily treats hair loss through vasodilation and growth-factor-related mechanisms [27], while preclinical evidence regarding potential reproductive effects remains limited [17]. In the present analysis, minoxidil generated fewer reports than finasteride and dutasteride in both databases. Given the small number of reports, this finding should be interpreted cautiously.

To explore biological features potentially related to the observed associations, this study conducted multi-level bioinformatic analyses. The overlap analysis prioritized HIF1A as a candidate linking drug-associated target lists with infertility-related testicular expression changes; however, this database-level overlap does not demonstrate a common downstream target or a drug-specific mechanism. HIF1A is a core transcriptional regulator of cellular responses to hypoxia and plays an important role in energy metabolism and oxygen homeostasis [28,29]. However, HIF1A is also a broadly responsive regulator of hypoxia, metabolism, inflammation, and tissue stress; therefore, its identification in infertility-related transcriptomes is biologically plausible but not drug-specific. The bulk and single-cell analyses were based on male-infertility datasets rather than drug-exposure models. Accordingly, these findings provide biological context for hypothesis generation but do not demonstrate that finasteride, dutasteride, or minoxidil induced HIF1A expression or that HIF1A mediated the reported adverse events. Drug-exposure models and functional experiments are required to test these possibilities.

Previous studies have implicated HIF1A-related hypoxic responses in male reproductive dysfunction. Under hypoxic conditions, HIF1A-dependent transcriptional responses have been associated with germ-cell apoptosis and reduced cellular proliferation [30]. Potential relationships between HIF1A expression, metabolic disturbance, and autophagy-related processes have also been discussed in the context of impaired sperm function. In patients with varicocele-associated oligoasthenozoospermia, progressive sperm motility was reported to be inversely correlated with HIF1A expression [31]. Sperm motility may also be regulated by seminal-plasma exosome-mediated signaling pathways [32]. These studies provide biological context for the higher HIF1A expression observed in the infertility-related testicular transcriptomic datasets analyzed here. However, the present analyses do not demonstrate abnormal activation of HIF1A or establish that HIF1A directly mediates oxidative stress, autophagy, or impaired spermatogenesis.

The cell-type-resolved analysis showed higher HIF1A expression in late spermatocytes, Leydig cells, and myoid cells in infertility samples. This pattern is compatible with the broad expression of HIF-1α in the male reproductive system and its response to testicular hypoxia [33–35]. Experimental studies have linked HIF1A to germ-cell apoptosis and suppression of steroidogenic activity in Leydig cells [36,37]. These studies provide biological context for our observations; however, the cross-sectional transcriptomic data do not establish that HIF1A directly caused the spermatogenic or endocrine abnormalities in the analyzed patients.

At the pathway level, CellChat analysis showed group-dependent inferred communication patterns across selected growth-factor-related ligand–receptor axes. The functional enrichment analyses identified a range of cellular, metabolic, mitochondrial, and protein-homeostasis-related processes, while GSEA showed that higher HIF1A expression was associated with immune-response-, wound-healing-, and cell-adhesion-related features. By contrast, lower HIF1A expression was associated with spermatid development, spermatid differentiation, and cilium- or flagellum-dependent motility. Previous studies have implicated hypoxia- and VEGF-related responses in testicular function and germ-cell development [30,38,39]. However, these literature findings provide biological context only. The CellChat and enrichment results obtained in the present study are inferential and associative and do not demonstrate increased pathway activity, HIF1A-mediated regulation, or a drug-specific mechanism.

Higher HIF1A expression was also accompanied by enrichment of immune-response- and cell-adhesion-related gene sets and positive correlations with several ssGSEA-derived immune-cell signature scores. Previous studies have linked HIF1A-related responses to inflammatory and tissue-remodeling processes [30,40]. Mast-cell accumulation, local immune dysregulation, blood–testis barrier disruption, and interstitial immune-cell infiltration have also been reported in male infertility-related conditions [41–43]. These findings provide biological context for the observed transcriptomic associations. However, ssGSEA-derived scores do not directly measure immune-cell abundance, and the correlations observed here do not establish that HIF1A directly drives testicular inflammation, tissue remodeling, barrier disruption, or infertility.

Clinical implications should be interpreted cautiously. Because disproportionality analyses from spontaneous reporting systems indicate reporting signals rather than incidence or causality, these findings do not by themselves justify changes to prescribing or labeling. For reproductive-age males—particularly those actively attempting conception or presenting with fertility concerns—clinicians may consider discussing the uncertainty of the current evidence, documenting baseline reproductive history, and obtaining a baseline semen analysis when clinically indicated. These considerations should support individualized counseling rather than be interpreted as a universal standard of care. Prospective studies with indication-, dose-, and route-stratified designs, together with experimental validation, are required before making definitive clinical or regulatory recommendations.

A key limitation is potential confounding by indication, dose, and route of administration. Although available administration-route information was summarized descriptively, indication and exposure details were frequently missing or incomplete, precluding definitive attribution of individual reports to AGA treatment. In particular, differences in systemic exposure across oral, topical, and other reported routes could influence spontaneous-reporting patterns. The findings should therefore be interpreted as hypothesis-generating reporting signals and require confirmation in prospective studies with indication-, dose-, and route-resolved exposure information.

Strengths of this study include the comparison of reporting patterns across two large spontaneous-reporting systems, the use of focused male fertility–related endpoints, and the integration of bulk and single-cell transcriptomic analyses to provide multidimensional exploratory bioinformatic findings. Nevertheless, several limitations should be acknowledged. Spontaneous-reporting systems are subject to reporting bias, underreporting, incomplete clinical information, indication confounding, and the absence of incidence denominators; therefore, disproportionality estimates cannot be interpreted as incidence rates, comparative risks, or causal effects. The transcriptomic analyses were based on relatively limited cross-sectional datasets, and the drug-associated target lists included curated, predicted, and indirect database relationships that may not represent direct molecular interactions. No drug-exposure models or functional experiments were performed to determine whether HIF1A contributes to reproductive phenotypes or whether the HIF1A-related findings are relevant to the investigated medications. In addition, the integrated workflow involved multiple sequential analyses, creating several opportunities for false-positive findings. Although Benjamini–Hochberg correction was applied within several individual analyses, no single procedure controlled the overall error rate across the full integrated workflow. Convergence across analyses should therefore be interpreted as exploratory rather than as independent confirmation of a biological mechanism. The HIF1A ROC result was derived from the analyzed discovery data without external validation and should likewise be interpreted cautiously. Overall, the HIF1A-related findings are hypothesis-generating and require confirmation using independent datasets and appropriately designed experimental models.

Future studies should use prospective designs with detailed indication, dose, route, treatment duration, and reproductive-outcome information to evaluate the long-term reproductive safety of these medications. Further investigation is particularly needed for minoxidil because the available clinical evidence and the number of relevant spontaneous reports remain limited. Drug-exposure models, animal studies, and in vitro experiments are also required to test whether and how HIF1A contributes to reproductive phenotypes and whether any such relationship is relevant to the investigated medications.

In conclusion, by combining real-world pharmacovigilance data and bioinformatics analyses, this study systematically evaluated potential associations between commonly used male alopecia therapeutic agents and male reproductive health. Our findings provide new perspectives for clinical medication safety and point directions for subsequent research. The pharmacovigilance analyses identified fertility-related reporting signals for the investigated medications, whereas the transcriptomic analyses identified exploratory HIF1A-related features in male infertility datasets. These evidence layers do not establish causality or a shared drug-specific mechanism. Further epidemiologic and experimental studies are required to evaluate the reporting signals and the biological hypotheses generated in this study.

## Supporting information

S1 Fig Graphical abstract. (TIF)

S1 Table Two-by-two contingency table for disproportionality analysis. (DOCX)

S2 Table Calculation formulas and signal detection criteria for disproportionality measures. (DOCX)

S3 Table Marker genes used for testicular cell-type annotation in single-cell RNA-sequencing analysis. (XLS)

S4 Table Immune cell marker gene sets used for ssGSEA-based immune infiltration analysis. (CSV)

S5 Table Genes significantly correlated with HIF1A expression in the merged GSE45885 and GSE45887 testicular transcriptomic dataset. (XLS)
