# Supplementary material for: Androgenetic alopecia drugs and male infertility: Evidence from pharmacovigilance and testicular transcriptomics
Source: PLoS One. 2026 Sep 24;21(9):e0357154. doi: 10.1371/journal.pone.0357154 (PMC13606971; doi:10.1371/journal.pone.0357154)
Supplement: Supplementary file 1 [file pone.0357154.s002.docx]

Two-by-two contingency table for disproportionality analysis.

| **Item** | **Target adverse events reported** | **Other adverse events reported** | **Total** |
| --- | --- | --- | --- |
| **Target drugs** | a | b | a + b |
| **Other drugs** | c | d | c + d |
| **Total** | a + c | b + d | a + b + c + d |
