# Supplementary material for: Androgenetic alopecia drugs and male infertility: Evidence from pharmacovigilance and testicular transcriptomics
Source: PLoS One. 2026 Sep 24;21(9):e0357154. doi: 10.1371/journal.pone.0357154 (PMC13606971; doi:10.1371/journal.pone.0357154)
Supplement: Supplementary file 2 [file pone.0357154.s003.docx]

The principles of disproportionate measurement and the criteria for signal detection.

| Method | Calculation formula | Criteria |
| --- | --- | --- |
| ROR | $ROR=\frac{a / c}{b / d}$ | a ≥ 3  95%CI (lower limit) > 1 |
|  | $SE(lnROR)=\sqrt{\frac{1}{a}+\frac{1}{b}+\frac{1}{c}+\frac{1}{d}}$ |  |
|  | $95\%CI= e^{\ln\left( ROR \right)\pm1.96se}$ |  |
| PRR | $PRR=\frac{a / (a+b)}{c / (c+d)}$ | a ≥ 3  95%CI (lower limit) > 1 |
|  | $SE(lnPRR)=\sqrt{\frac{1}{a}-\frac{1}{a+b}+\frac{1}{c}-\frac{1}{c+d}}$ |  |
|  | $95\%CI= e^{\ln\left( PRR \right)\pm1.96se}$ |  |
|  | $\chi2 =\frac{{(ad-bc)}^{2}(a+b+c+d)}{( a+b)(a+c)(c+d)(b+d)}$ | a ≥ 3  PRR ≥ 2  $\chi2\geq4$ |
| BCPNN | IC=${log}_{2}a(a+b+c+d)/(\left( a+c \right)(a+b))$ | IC025>0 |
|  | $95\%CI= E\left( \mathrm{IC} \right)\pm2V(IC)^0.5$ |  |
| MGPS | $EBGM=\frac{a(a+b+c+d)}{\left( a+c \right)(a+b)}$ | EBGM05>2 |
|  | $SE(lnEBGM)=\sqrt{\frac{1}{a}+\frac{1}{b}+\frac{1}{c}+\frac{1}{d}}$ |  |
|  | $95\%CI= e^{\ln\left( EBGM \right)\pm1.96se}$ |  |

**Notes:** Equation: a, number of reports containing both the target drug and target adverse drug reaction; b, number of reports containing other drugs and the target adverse drug reaction; c, number of reports of the target drug but not the adverse drug reaction of other drugs; d, number of reports of other drugs and other adverse events.

**Abbreviations:** ROR, reporting odds ratio; PRR, proportional reporting ratio; BCPNN, Bayesian confidence propagation neural network; EBGM, empirical Bayesian geometric mean; IC, information component; SE, standard error; 95% CI, 95% confidence interval; IC025, the lower limit of 95% CI of the IC; EBGM05, the lower limit of 95% CI of the EBGM; E(IC), the IC expectations; V(IC), the variance of IC; χ², chi-squared。
